# Binding of dihydromyricetin and its metal ion complexes with bovine serum albumin

**DOI:** 10.1080/13102818.2014.915077

**Published:** 2014-07-08

**Authors:** Qingquan Guo, Juan Yuan, Jinhua Zeng

**Affiliations:** ^a^ School of Chemical Engineering and Light Industry, Guangdong University of Technology, Guangzhou, P.R. China; ^b^ Guangdong Food Industry Institute, Guangzhou, P.R. China

**Keywords:** binding, bovine serum albumin, complexes, DMY, spectroscopy

## Abstract

The binding mechanisms of the interaction of three dihydromyricetin (DMY)–metal complexes (DMY–Cu (II) complex, DMY–Mn (II) complex, DMY–Zn (II) complex) and DMY with bovine serum albumin (BSA) were investigated using fluorescence and ultraviolet spectroscopy at different temperatures. The results indicated some differences in the binding process between different DMY–metal complexes and BSA compared with that of free DMY. All of the complexes and DMY quenched the fluorescence of BSA based on static mode combined with radiationless energy transfer, yet having different binding distance based on the Förster theory. Different DMY–metal complexes can change the binding constants. The binding constants increase for DMY–Cu (II) and DMY–Mn (II) complexes, whereas the opposite is true for the DMY–Zn (II) complex compared to the one with free DMY. The DMY–metal complexes can also affect the types of the interaction. The van der Waals forces and hydrogen bonding may play a major role in the interaction of free DMY with BSA, while for the three complexes, the nature of the binding forces lies in hydrophobic forces and hydrogen bonding based on the thermodynamic parameters.

## Introduction

Dihydromyricetin (3,5,7,3′,4′,5′-hexahydroxy-2,3-dihydroflavonol, DMY) is one of the too few flavonoids with a large-scale value, because its content in vine tea (a plant widely distributed in southern China) can be over 20% (w/w), and it has been proved to have multiple biological properties such as hypoglycemic, antithrombotic, antioxidant, enhancement immunity, anti-inflammatory, antibacterial activities, etc.[1,2] Meanwhile, DMY is a good metal-ion-chelating ligand due to its molecular structure with upper superdelocalizability, integral conjugated large π bond, strong coordinated oxygen atoms and appropriate spatial configuration.[2,3] Some DMY–metal complexes show better biological activity, such as antioxidant and anticancer activity, than free DMY or the metal itself.[4,5] However, the activities of different DMY–metal complexes *in vivo* remain uncertain, and questions concerning their transport, absorption, metabolism and bioavailability *in vivo* are still unanswered.

Serum albumin is the major transport protein in the blood circulatory system (accounting for 52%–60% of the total plasma protein). As a drug carrier, the functions of serum albumin are binding and transporting of various endogenous and exogenous small molecules (including drugs) to specific organs.[6,7] Drug–protein interactions in the bloodstream are known to induce conformational changes to the protein, simultaneously affecting the distribution, free concentration and the metabolism of drugs.[8,9] The study of the interaction between drugs and serum albumin is not only important in providing salient information about the nature of drugs and pharmacokinetics, but also helpful to explain the relationship between the structures and functions of drugs.[10] Therefore, the studies of the interactions of drug–serum albumin are very essential in drug development.

At present, there have been some reports about the binding process between DMY and serum albumin, and the effect of some common metal ions such as Cu^2+^, Ca^2+^, Mg^2+^, Zn^2+^, Ni^2+^, K^+^, Co^2+^ and Fe^3+^ on the binding constant between DMY and bovine serum albumin (BSA).[11–13] However, little information has been obtained about the binding process of different DMY–metal complexes with serum albumin when DMY chelates metal ions to form metal complexes, and about some differences in the binding process for different DMY–metal complexes in comparison to the complex with free DMY.

In this study, ultraviolet–visible (UV–vis) and fluorescence spectrometry methods are used to study the interactions of three DMY–metal complexes: DMY–Cu (II), DMY–Mn (II) and DMY–Zn (II) complex with BSA compared to that of free DMY. The BSA has been selected as the protein model because of its structural homology with human serum albumin, low cost and the elements Cu, Mn and Zn because they are all important to the body's physiological activities. This study provides insight into the possibilities of transport, disposition and efficiency of DMY–metal complexes in blood plasma, thus providing theoretical basis for future drug design.

## Materials and methods

All fluorescence spectra were measured on an RF-5301 fluorescence spectrophotometer (Shimadzu, Japan) equipped with a SB-11 water bath (Eyela, Japan) and 1.0 cm quartz cells. The widths of both the excitation slit and emission slit were set at 3.0 nm. The UV–vis spectra were recorded on a UV-2450 UV–vis spectrophotometer (Shimadzu, Japan).

BSA and DMY (Purity > 98.0%) were purchased from Sigma (Sigma Chemical Co., St. Louis, MO, USA). The stock solution of BSA was prepared by dissolving BSA in Tris-HCl buffer solution (0.1 mol L^−1^ Tris-HCl/0.1 mol L^−1^ NaCl, pH 7.4). DMY–Cu (II) complex ([C_15_H_l0_O_8_Cu]·H_2_O), DMY–Mn (II) complex ([C_15_H_10_O_8_Mn·2H_2_O]·2H_2_O) and DMY–Zn (II) complex ([C_15_H_l0_O_8_Zn]·2H_2_O) were obtained from our previous study.[14] Concentrated stock solutions of DMY and its metal complexes were prepared by dissolving them in a small amount of dimethyl sulphoxide, then diluting with Tris-HCl buffer solution to get the required concentrations. All other reagents and solvents were of analytical grade and used without further purification, and aqueous solutions were prepared using freshly double-distilled water.

### Preparation of DMY–Cu (II)/DMY–Mn (II)/DMY–Zn (II) complex

The solid DMY (10 mmol) was dissolved into 60 mL of ethanol (95%, w/w). Then the pH of the solution was adjusted to 7–8 with sodium carbonate. After 5 min, (CH_3_COO)_2_Cu·H_2_O/(CH_3_COO)_2_Mn·4H_2_O/(CH_3_COO)_2_Zn·2H_2_O (10 mmol) was added to the above mixture separately. After being stirred and heated to reflux for 8 h at 70 °C, the reaction mixture was cooled to room temperature. The mixture was filtered and repeatedly washed with ethanol and water. The solid product was dried under vacuum for 48 h at room temperature.[14] The products were examined by UV–vis spectroscopy, Fourier transform infrared spectroscopy, element analysis and thermal gravimetric analysis, and their molecular structure is: [C_15_H_l0_O_8_Cu]·H_2_O for the DMY–Cu (II) complex, [C_15_H_10_O_8_Mn·2H_2_O]·2H_2_O for the DMY–Mn (II) complex and [C_15_H_l0_O_8_Zn]·2H_2_O for the DMY–Zn (II) complex.

### Fluorescence spectra

A quantitative analysis of the potential interaction between DMY or its complexes and BSA was performed by fluorimetric titration. In each titration, the fluorescence spectrum was measured with BSA concentration of 1.0 × 10^-6^ mol L^−1^, then titration was done by successive addition of DMY or its complexes (0.0–7.0 × 10^−5^ mol L^−1^). The fluorescence emission spectra were recorded at two different temperatures (300 and 310 K) in the wavelength range of 300–450 nm with excitation wavelength of 280 nm.

The spectra were corrected for inner filter effect that refers to the absorption of light at the excitation and/or emission wavelength by a compound present in a solution. The following equation was used for correction:[15](1) 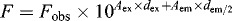
where *F*_obs_ is the measured fluorescence, *F* the correct fluorescence intensity that would be measured in the absence of inner filter effect, *d*_ex_ = 1 cm and *d*_em_ = 0.4 cm are the cuvette pathlength in the excitation and emission direction, respectively and A_ex_ and A_em_ are the measured change in the absorbance value at the excitation and emission wavelength, respectively, caused by ligand addition. The experiments were repeated and found to be reproducible within experimental errors (<1%).

### Uv–vis absorption spectra

The absorption spectra of DMY or its complexes with a concentration of 2.0 × 10^−5^ mol L^−1^ were measured in the range of 200–400 nm at 300 K.

The absorption spectra of BSA in the presence of different concentrations of DMY or its metal complexes were also recorded in the range of 200–350 nm at 300 K. The concentration of BSA was kept at 5.0 × 10^−6^ mol L^−1^, while the concentrations of DMY or its complexes were varied from 0.0–5.0 × 10^-5^ mol L^−1^.

## Results and discussion

Spectrometry has been widely used in the research of small molecule drugs and protein interaction because of its high sensitivity, high selectivity and requirement for a lesser amount of sample. Fluorescence spectroscopy is the most common technique used in the study of the interaction of small molecule drugs with protein; meanwhile, UV-vis absorption spectroscopy is a very simple but very effective method to explore the interaction between small molecule drugs and protein.[16] That is why, we chose these two methods to study the mechanism between different metal–DMY complexes or free DMY and BSA.

### Fluorescence quenching

Fluorescence quenching is the decrease of the quantum yield of fluorescence from a fluorophore induced by a variety of molecular interactions such as excited-state reactions, molecular rearrangements, ground-state complex formation, collisional quenching and energy transfer.[16] The mechanisms of quenching are usually classified as either dynamic quenching or static quenching. Dynamic quenching results from collision between the fluorophore and a quencher, while static quenching results from non-fluorescent complex formation in the ground state of the fluorophore. Dynamic and static quenching can be distinguished by their different dependence on temperature and viscosity, or preferably by lifetime measurements. For dynamic quenching, higher temperatures result in faster diffusion and larger amounts of collisional quenching, thus the quenching constant increases with increasing temperature. However, the reverse effect would be observed for static quenching.[17,18]

BSA has three intrinsic fluorophores: tryptophan, tyrosine and phenylalanine. Of these, tryptophan is mostly responsible for the intrinsic fluorescence of BSA, as the quantum yield of phenylalanine in BSA is very low and the fluorescence of tyrosine is almost totally quenched by drugs. When the fluorescence emission spectra of BSA are measured with a series of concentrations of quencher at a fixed excitation wavelength of 280 nm, the fluorescence emission peak of BSA at 340 nm gives the information of tryptophan residues.[19]

**Figure 1.**
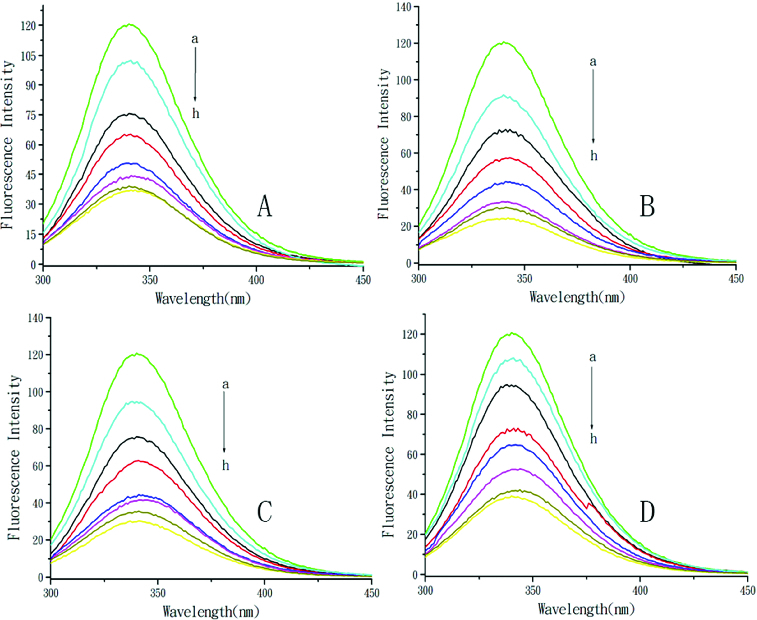
Quenching effect of DMY and its complexes on BSA fluorescence intensity. Different concentrations of DMY (A), DMY–Cu (II) (B), DMY–Mn (II) (C) and DMY-Zn (II) (D): a–h: 0.0 × 10^−5^ mol L^−1^ (a), 1.0 × 10^−5^ mol L^−1^ (b), 2.0 × 10^−5^ mol L^−1^ (c), 3.0 × 10^−5^ mol L^−1^ (d), 4.0 × 10^−5^ mol L^−1^ (e), 5.0 × 10^−5^ mol L^−1^ (f), 6.0 × 10^−5^ mol L^−1^ (g), 7.0 × 10^−5^ mol L^−1^ (h); λ_ex_ = 280 nm; *T* = 300 K.

The fluorescence spectra of BSA with the addition of DMY or its metal complexes are shown in Figure 1. With the addition of DMY or complexes, the fluorescence intensity of BSA remarkably decreased with a slight red shift of the maximum emission wavelength, which indicated that DMY or its complexes could interact with BSA and quench its intrinsic fluorescence. This suggests that the micro-environment of the tryptophan residue in BSA was changed and DMY or its complexes were situated in close proximity to the tryptophan residues for quenching to occur. The DMY–Cu (II) complex and the DMY–Mn (II) complex could quench the fluorescence of BSA to a greater extent than the DMY–Zn (II) complex as compared to DMY alone. The results demonstrated that Cu^2+^, Mn^2+^ and Zn^2+^ chelation of DMY could affect the quenching effects of free DMY on BSA fluorescence.

**Figure 2.**
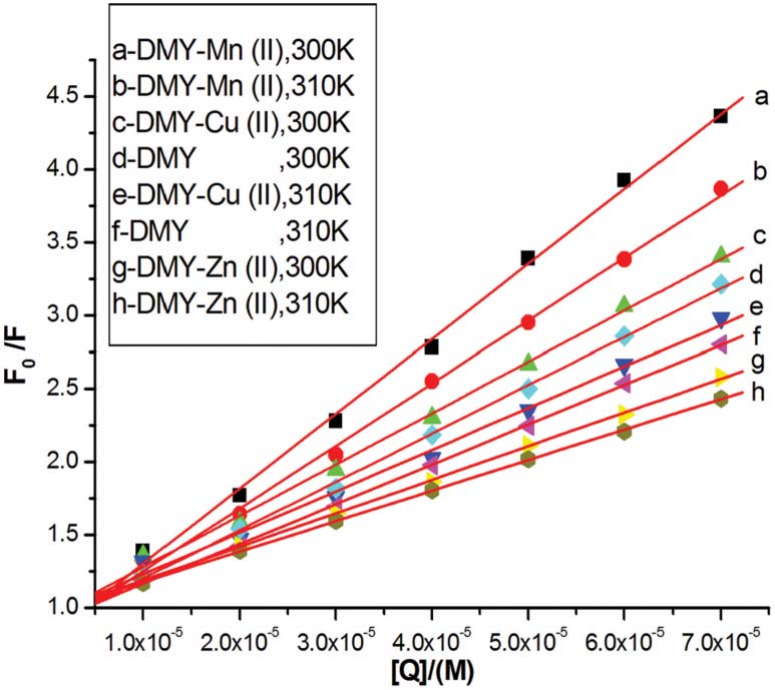
Stern-Volmer plots for BSA fluorescence quenching by DMY, DMY-Cu (II), DMY-Mn (II) and DMY-Zn (II) complexes at 300 and 310 K.

To elucidate the fluorescence quenching mechanism, the fluorescence quenching data were analysed using the Stern–Volmer equation [16] as follows:(2) 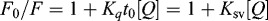
where *F*_0_ and *F* are the correct fluorescence intensities of BSA in the absence and presence of the quencher, respectively; *K*_sv_ is the Stern–Volmer dynamic quenching constant; *K_q_* is the quenching rate constant of the biomolecule (*K_q_* = *K*_sv_/τ_0_), τ_0_ is the average lifetime of the fluorophore in the absence of the quencher (τ_0_ = 10^−8^ s),[20] and [*Q*] is the concentration of the quencher. The curves of *F*_0_/*F* versus [*Q*] at two different temperatures (300 and 310 K) are displayed in Figure 2. To determine the quenching type, the effects of temperature on the interactions of DMY or its complexes with BSA were studied. The corresponding *K*_sv_ values for the interaction between DMY or its complexes and BSA are shown in Table 1. The results show that all *K*_sv_ values decrease with increasing temperature, and the values of *K_q_* are much greater than the limiting diffusion rate constant of the biomolecule (2.0 × 10^10^ L mol^−1^ s^−1^),[20,21] which suggests that the quenching mechanism of BSA by DMY and its complexes is not initiated by dynamic quenching but probably by static quenching. Therefore, the metal complexes of DMY do not change the quenching type of DMY.

**Table 1.** Stern-Volmer quenching constants (*K*_sv_) and quenching rate constants (*K_q_*) for the interactions of DMY, DMY–Cu (II) complex, DMY–Mn (II) complex and DMY–Zn (II) complex with BSA.

*T* (K)	Compounds	*K*_sv_ (×10^4^ L mol^−1^)	*K_q_* (×10^12^ L mol^−1^ s^−1^)	*R*^2^
300	DMY	3.17	3.17	0.9973
310		2.79	2.79	0.9992
300	DMY-Cu (II)	3.22	3.22	0.9932
310		2.89	2.89	0.9961
300	DMY-Mn (II)	5.44	5.44	0.9974
310		4.56	4.56	0.9965
300	DMY-Zn (II)	2.39	2.39	0.9967
310		2.08	2.08	0.9992

### UV–vis spectra

**Figure 3.**
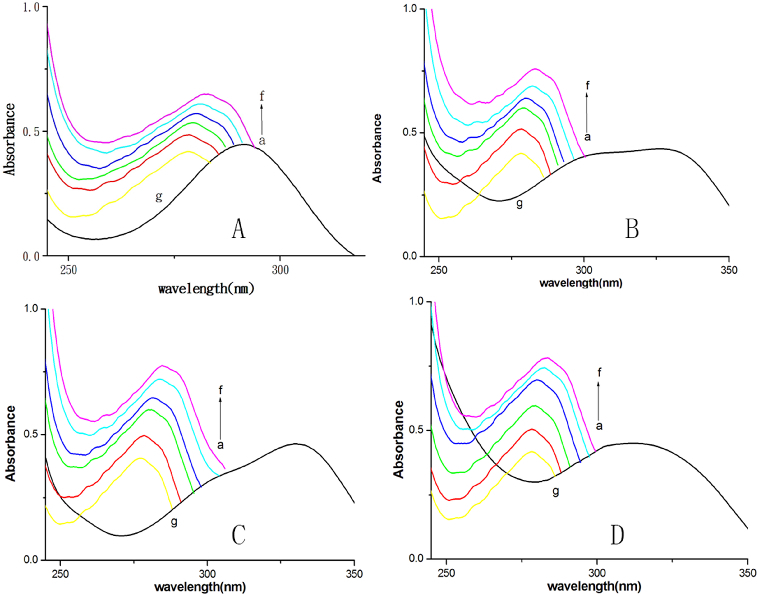
UV-vis spectra of DMY (A) or its complexes (B–D) in the presence (a–f) or absence of BSA (g). Different concentrations of DMY (A), DMY-Cu (II) (B), DMY-Mn (II) (C) and DMY-Zn (II) (D): 0.0 × 10^−5^ mol L^−1^ (a), 1.0 ×1 0^−5^ mol L^−1^ (b), 2.0 × 10^−5^ mol L^−1^ (c), 3.0 × 10^−5^ mol L^−1^ (d), 4.0 × 10^−5^ mol L^−1^ (e), 5.0 × 10^−5^ mol L^−1^ (f); *C*_BSA_ = 5.0 × 10^−6^ mol L^−1^. In the absence of BSA (g), *C*_DMY_ = *C*_DMY-Cu (II)_ = *C*_DMY-Mn (II)_ = *C*_DMY-Zn(II)_ = 2.0 × 10^−5^ mol L^−1^.

To further confirm that the quenching mechanism of fluorescence of BSA by DMY or its complexes is static quenching, UV–vis absorption spectra were obtained. Most proteins with tryptophan residues have an absorption peak at near 280 nm. After the drugs are added to a protein solution, a red shift of absorption peaks and a change of absorbance are observed in the absorption spectra of the protein if it binds to small molecule drugs.[11,22] The absorption spectra of BSA in the presence and absence of DMY and its complexes (Figure 3) showed that, with the addition of DMY or its complexes, the absorbance intensity of BSA increases and the maximum absorption peaks shift towards the longer wavelength region. This indicates that BSA can bind to DMY or its complexes to form a new complex. As static quenching results from non-fluorescent complex formation, this result (Figure 3) also reconfirms that there is static quenching in the interaction processes of DMY or its complexes with BSA.

### Binding constants (*K_b_*) and number of binding sites (*n*)

The level of drug binding with serum albumin is critical and directly correlates with the transport, disposition and efficacy of the drug *in vivo*. If a drug has low ability to bind with serum albumin, the amount of drug available to diffuse into the target tissue may be significantly reduced, and then the efficacy of the drug may be poor and vice versa.

For static quenching interaction, the binding constant (*K_b_*) and the number of binding sites (*n*) can be obtained from the equation as follows:[23](3) 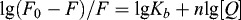
where *F*_0_ and *F* represent the correct fluorescence intensities of BSA in the absence and in the presence of quencher, *K_b_* is the binding constant, *n* is the number of binding sites per BSA and [*Q*] is the concentration of the quencher. Based on Equation (3), the values of *K_b_* and *n* can be obtained using the double-logarithm algorithm curve. Table 2 lists the corresponding calculated binding constants and the number of binding sites for DMY and its complexes. The correlation coefficients are larger than 0.993, indicating that the assumptions underlying the derivation of Equation (3) are reasonable.

**Table 2.** Binding parameters and relative thermodynamic parameters for the interaction of DMY, DMY–Cu(II) complex, DMY–Mn (II)complex and DMY–Zn(II) complex with BSA.

*T* (K)	Compounds	*K_b_* (L·mol^−1^)	*n*	*R*^2^	*ΔH* (kJ·mol^−1^)	*ΔG* (kJ·mol^−1^)	*ΔS* (J·mol^−1^·K^−1^)
300	DMY	1.30 × 10^5^	1.11	0.9968	−31.89	−29.37	−8.39
310		8.62 × 10^4^	1.05	0.9992		−29.29	
300	DMY-Cu (II)	2.95 × 10^5^	1.24	0.9938	−29.67	−31.41	5.80
310		2.01 × 10^5^	1.21	0.9946		−31.47	
300	DMY-Mn (II)	2.13 × 10^5^	1.15	0.9969	−27.68	−30.60	9.73
310		1.49 × 10^5^	1.13	0.9947		−30.70	
300	DMY-Zn (II)	7.76 × 10^4^	0.97	0.9960	−24.95	−28.08	10.45
310		5.62 × 10^4^	0.94	0.9991		−28.19	

The calculated *K_b_* values suggest that moderate affinity exists between BSA and DMY, DMY–Cu (II) complex, DMY–Mn (II) complex and DMY–Zn (II) complex, and the values of *K_b_* decrease the higher the temperature is, indicating that the capacity of DMY or its complexes to bind to BSA decreases. Increasing the temperature results in an increase in the diffusion coefficient and a reduction in the stability of the BSA–DMY/BSA–DMY–Cu (II)/BSA–DMY–Mn (II)/BSA–DMY–Zn (II) complex.

BSA contains hydrophobic groups in the interior of the tertiary structure and polar groups such as amino, hydroxyl and sulphydryl groups at the surface. Hydrogen bonding may take place between hydrogen atoms in DMY or its complexes and polar groups at the BSA surface. As shown in Table 2, the *K_b_* values increase in this order: DMY–Zn (II) < DMY < DMY–Mn (II) < DMY–Cu (II). The lowest binding constant is that of DMY–Zn (II) and the highest one is that of DMY–Cu (II) among the four studied compounds. The reason may be that in the preparation of DMY–Zn complex, Zn^2+^ chelation to DMY reduces one of the hydroxyl groups in DMY, which weakens the possibility of formation of hydrogen bonds between the DMY–Zn (II) complex and BSA; in addition, Zn^2+^ chelation increases the molecular size and hydrophobility, which weakens the capacity of the DMY–Zn (II) complex to penetrate into the tryptophan hydrophobic regions of BSA. Thus, the DMY–Zn(II) complex reduces the binding affinity to BSA. Another reason may be the existence of synergistic action about DMY and Mn^2+^ or Cu^2+^ binding with BSA; therefore, DMY–Cu (II) complex and DMY–Mn (II) complex increase the binding affinity to BSA. In other words, compared with DMY, the decreasing of the binding constant would shorten the retention time of DMY–Zn (II) in the blood and would facilitate the easy release of the drug, indicating that the transporting ability of DMY–Zn (II) in the blood may be significantly reduced and the efficacy may be consequently poor. The DMY–Cu (II) and DMY–Mn (II) complexes increase the affinities to serum albumin and would improve the transporting ability of serum albumin for them. The increasing of the binding constant would lengthen the retention time of DMY–Cu (II) and DMY–Mn (II) complexes in the blood, and thus extend the efficacy. Therefore, metal ion chelation of DMY can change the transport, disposition and pharmacological effects in comparison with free DMY. The values of *n* are approximately equal to 1, suggesting that there is one binding site on BSA for DMY, DMY–Cu (II) complex, DMY–Mn (II) complex and DMY–Zn (II) complex. All of the values of *K_b_* were proportional to the binding sites (*n*) with a high correlation coefficient, which confirmed that the mathematical model used in the experiment was suitable to study the interaction between DMY or its complexes and BSA.

### Binding mode

The thermodynamic parameters for protein interactions can give information about the main forces contributing to protein stability. Basically, four main types of interactions: hydrogen bonds, electrostatic forces, van der Waals forces and hydrophobic forces, play critical roles in the interactions between small molecules and macromolecules.[20] To characterize the force between DMY or its complexes and BSA, thermodynamic parameters on the temperatures were analysed. The thermodynamic parameters, enthalpy change (Δ*H* ^o^), entropy change (Δ*S* ^o^) and free energy change (Δ*G* ^o^) were evaluated using the following equations:[24,25](4) 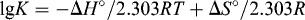

(5) 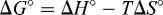
where *K* and *R* are the binding constant and gas constant, respectively; *T* is the experimental temperature. The thermodynamic parameters are summarized in Table 2.

A positive Δ*S*° change occurs when the water molecules are arranged in an orderly fashion around the ligand and the protein acquires a more random configuration as a result of hydrophobic interactions, whereas a negative value of Δ*S*° suggests that the binding process is predominately enthalpy driven by means of hydrogen binding and van der Waals forces interactions.[17] In the present study, the negative Δ*H* ^o^ value may not be attributed to electrostatic interactions, as higher negative Δ*H* ^o^ value would be observed whenever there is hydrogen bonding.[25,26] Generally, the interaction of small molecules with proteins does not involve only a certain kind of action force, but a combined action with other action forces. Therefore, from the data given in Table 2, it could be suggested that both hydrophobic interactions and hydrogen bonds play a major role in the binding of DMY–metal complexes to BSA, and both van der Waals forces and hydrogen bonding play a significant role in the binding of free DMY to BSA. The results indicate that the metal complexes of DMY can affect the type of interactions between DMY and protein to some extent.

### Energy transfer between BSA and DMY

**Figure 4.**
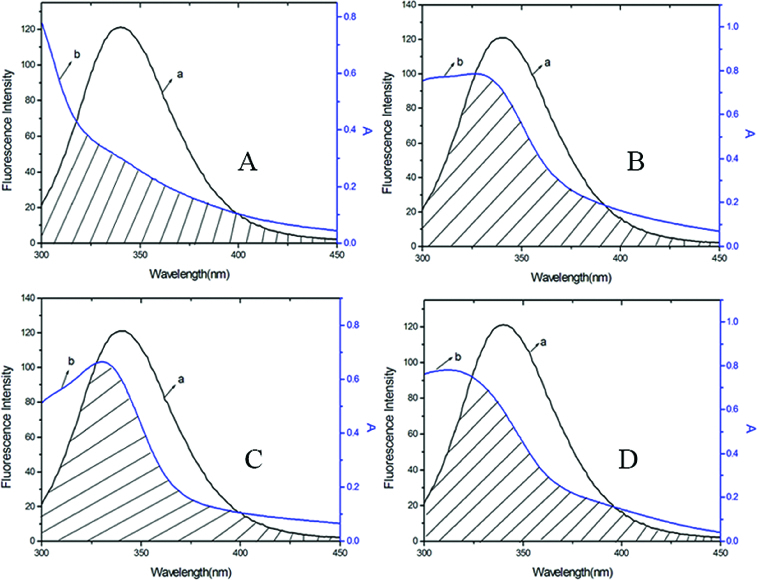
Overlap of the fluorescence emission spectrum of BSA (a) and the UV–vis absorption spectra (b) of DMY (A), DMY-Cu (II) (B), DMY-Mn (II) (C) and DMY-Zn (II) (D). *C*_BSA_ = 1.00 × 10^−6^ mol L^−1^, *C*_DMY_ = *C*_DMY-Cu (II)_ = *C*_DMY-Mn (II)_ = *C*_DMY-Zn(II)_ = 3.0 × 10^−5^ mol L^−1^.

The Förster resonance energy transfer theory is an important method to investigate a variety of biological phenomena such as energy transfer processes. According to this theory, energy transfer could happen under the following conditions: (1) the donor and the acceptor dipoles have favourable relative orientation; (2) the fluorescence emission spectrum of the donor and the absorption spectrum of the acceptor overlap to some extent and (3) the distance between the donor and the acceptor is <8 nm.[27,28] Here in this study, the donor and the acceptor are BSA and DMY or its complexes, respectively. The overlap of the absorption spectra of DMY, DMY–Cu (II) complex, DMY–Mn (II) complex, DMY–Zn (II) complex, and the fluorescence emission spectrum of BSA at 300 K are shown in Figure 4.

The efficiency of energy transfer (*E*) was determined by Förster's energy transfer theory,[27](6) 
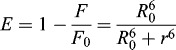
where *F* and *F*_0_ are the fluorescence intensities of BSA with or without the existence of ligand, respectively; *r* is the distance between acceptor and donor and *R*_0_ is the critical distance, which is evaluated as follows when the transfer efficiency is 50%:[28](7) 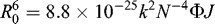
where *k* ^2^ is the spatial orientation factor of the dipole, *N* is the refractive index of the medium, Φ is the fluorescence quantum yield of the donor. For ligand–BSA interaction, *k* ^2^ = 2/3, *N* = 1.36 and Φ = 0.15.[17,21] *J* is the overlap integral of the fluorescence emission spectrum of the donor and the absorption spectrum of the acceptor, where *J* is given by the equation:[29,30](8) 
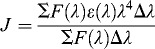
where *F*(λ) is the fluorescence intensity of the donor in the wavelength range λ to λ + Δλ and *ϵ*(λ) is the molar absorption coefficient of the acceptor at wavelength λ.

As shown in Table 3, the donor-to-acceptor distances for DMY, DMY–Cu (II) complex, DMY–Mn (II) complex and DMY–Zn (II) complex are 3.322, 3.120, 3.201 and 3.637 nm, respectively. All of the *r* values are much smaller than 8 nm and in the range of 0.5 to 1.5 *R*_0_, which suggests that radiationless energy transfer from BSA to DMY or its complexes occurs with high possibility. The larger *r* value compared to *R*_0_ also reveals the presence of a static type of quenching mechanism. Therefore, the quenching mechanism for DMY, DMY–Cu (II) complex, DMY–Mn (II) complex and DMY–Zn (II) complex to BSA is static quenching combined with radiationless energy transfer. Moreover, the stronger the binding capacity between a compound and BSA is, the smaller the distance between the complexes and BSA is. Hence, the binding capacity of DMY and its complexes to BSA is: DMY–Zn (II) < DMY < DMY–Mn (II) < DMY–Cu (II), which is consistent with the above results.

**Table 3.** Parameters of energy transfer between DMY, DMY-Cu (II) complex, DMY-Mn (II) complex, DMY-Zn (II) complex and BSA.

Compounds	*J* (cm^3^·L·mol^−1^)	*E*	*R*_0_ (nm)	*r* (nm)
DMY	1.34 × 10^−14^	0.178	2.570	3.322
DMY-Cu (II)	1.57 × 10^−14^	0.270	2.643	3.120
DMY-Mn (II)	1.52 × 10^−14^	0.235	2.627	3.201
DMY-Zn (II)	1.48 × 10^−14^	0.122	2.617	3.637

Hajji et al. [31] and Kang et al. [32] have performed similar studies on the chelation of quercetin with iron and copper, and on the interaction of coordination compounds of Cd (II), Co (II), Cu (II) and Zn (II) with rutin. These reports show that the interaction mechanisms of the coordination of different metals and different flavones are different. The research on the interaction between DMY or its metal complexes with BSA may potentiate their application in drug design, and in turn, may provide some specific insight into their regulatory roles in biological systems.

## Conclusions

In this study, the interactions between BSA and DMY–Cu (II) complex, DMY–Mn (II) complex and DMY–Zn (II) complex compared with that of free DMY were studied by fluorescence spectroscopy and UV–vis spectroscopy at different temperatures. The fluorescence of BSA was shown to be quenched by DMY and its complexes all through static mode and radiationless energy transfer. The binding constants for BSA are 7.74 × 10^4^ L mol^−1^ with DMY–Zn (II), 1.30 × 10^5^ L mol^−1^ with DMY, 2.13 × 10^5^ L mol^−1^ with DMY–Mn (II) and 2.95 × 10^5^ L mol^−1^ with DMY–Cu (II) at 300 K. It was demonstrated that metal complexes of DMY can affect the type of interactions between DMY and protein; hydrophobic forces and hydrogen bonds may play a major role in the interaction of DMY with BSA, while for the three DMY–metal complexes, the binding modes include van der Waals forces and hydrogen bonding based on the thermodynamic parameters. The differences in the binding constant, binding mode and binding distance for the interaction of different DMY–metal complexes with BSA in comparison with that of free DMY, indicate that different DMY–metal complexes can change the transport, disposition and pharmacological effects of free DMY, and can provide an important theoretical support for the future development of new anticancer drugs.
